# Using research to influence sexual and reproductive health practice and implementation in Sub-Saharan Africa: a case-study analysis

**DOI:** 10.1186/1478-4505-9-S1-S10

**Published:** 2011-06-16

**Authors:** Olivia Tulloch, Philippe Mayaud, Yaw Adu-Sarkodie, Baafuor Kofi Opoku, Nana Oye Lithur, Eugene Sickle, Sinead Delany-Moretlwe, Mwita Wambura, John Changalucha, Sally Theobald

**Affiliations:** 1 International Health Research Group, Liverpool School of Tropical Medicine, Pembroke Place, Liverpool, L3 5QA, UK; 2 London School of Hygiene & Tropical Medicine, London, UK; 3 School of Medical Sciences, Kwame Nkrumah University of Sciences & Technology, Kumasi, Ghana; 4 INDEPTH Network, Ghana; 5 The Reproductive Health and HIV Research Unit, University of the Witwatersrand, South Africa; 6 National Institute for Medical Research, Mwanza Centre, Tanzania

## Abstract

**Background:**

Research institutions and donor organizations are giving growing attention to how research evidence is communicated to influence policy. In the area of sexual and reproductive health (SRH) and HIV there is less weight given to understanding how evidence is successfully translated into practice. Policy issues in SRH can be controversial, influenced by political factors and shaped by context such as religion, ethnicity, gender and sexuality.

**Methods:**

The case-studies presented in this paper analyse findings from SRH/HIV research programmes in sub-Saharan Africa: 1) Maternal syphilis screening in Ghana, 2) Legislative change for sexual violence survivors In Ghana, 3) Male circumcision policy in South Africa, and 4) Male circumcision policy in Tanzania. Our analysis draws on two frameworks, Sumner et al’s synthesis approach and Nutley’s research use continuum.

**Results:**

The analysis emphasises the relationships and communications involved in using research to influence policy and practice and recognises a distinction whereby practice is not necessarily influenced as a result of policy change – especially in SRH – where there are complex interactions between policy actors.

**Conclusion:**

Both frameworks demonstrate how policy networks, partnership and advocacy are critical in shaping the extent to which research is used and the importance of on-going and continuous links between a range of actors to maximize research impact on policy uptake and implementation. The case-studies illustrate the importance of long-term engagement between researchers and policy makers and how to use evidence to develop policies which are sensitive to context: political, cultural and practical.

## Background

The extent to which research evidence plays a part in of health policy and implementation varies considerably; both donors and researchers recognize how findings are communicated as an important factor. The often highly politicised nature of sexual and reproductive health (SRH) and HIV issues complicates the factors influencing policy development and implementation. It is an arena which has a high degree of civil society participation and touches upon sensitive religious, cultural, and social aspects of people’s lives. For the purposes of this paper we define health practice as the uptake and implementation of national or international health guidelines and policies by the formal health sector; we aim to illustrate that there are multiple factors, beyond uptake of research evidence into policy, which influence health practices in SRH and HIV (henceforth referred to in short as ‘SRH’).

Analytical theories and models on getting research into policy and practice (GRIPP) abound (see Sumner et al, [1]). Most highlight policy processes rather than practice; see for example the ODI RAPID framework [2] and Walt and Gilson’s policy triangle [3] which illustrates how actors, processes and context interact to shape policy content. Alternative models merge policy and practice [4] without differentiating between the policy or practice processes and dynamics at play. The tendency in the existing models is to give less weight to analysing the influences on practice, or at least not to draw a distinction between influences on policy *and* practice. Networks of actors across the policy continuum are frequently central to creating a link to practice (as well as policy); this paper argues that use of collaborative partnerships, media coverage, ‘knowledge brokers’ and advocacy as part of targeted communications strategies can forge the link between research evidence and policy implementation by connecting researchers with policy makers and practitioners [5,6].

**Figure 1:**
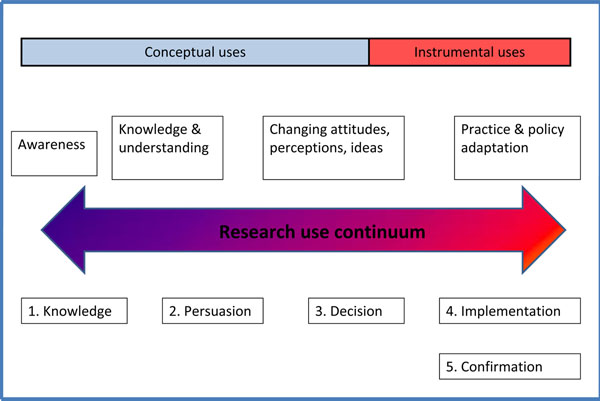
**Research use continuum.** Adapted from Nutley et al [4]

Several typologies have been constructed to explain the ways in which research may be used by both policy makers and practitioners. One simple distinction is between ‘instrumental’ and ‘conceptual’ research use which has been adapted by Nutley et al [4] to create a continuum of research evidence (Fig 1) upon which we draw upon in our analyses. Instrumental research use applies to the direct impact of research on policy and practice decisions. Conceptual research use applies to less demonstrable, subtle or indirect impact, such as influences on attitudes or understanding of policy makers and practitioners.

Nutley et al, (2007) argue that the instrumental and conceptual research uses can be viewed as a continuum. This illustrates the interactive processes often found in research use and their theory, and underscores the relative importance of conceptual research use alongside the more obviously demonstrable instrumental research use. This paper examines these types of research uses from four case-studies which explore the interactions of policy actors and processes along the SRH health practice continuum.

Health policy analyses have been criticised for a tendency to report single case-studies presenting results which are non-predictive and non-transferrable [7,8]. This is an inherent problem with policy analyses, events can be explained but the lessons learned may not be applicable to other contexts, nor able to present explanatory patterns. Multiple case-study design is arguably a solution: it is a research strategy for generalising the results of purposively selected cases, and its features complement each other to ensure valid inferences from the sample cases to the target population [9]. Published literature on health policy processes is dominated by authors based in Northern institutions [8]; the choice of case-studies here aims to give voice to researchers based in Southern institutions and to explore SRH research to policy and practice interactions in Sub-Saharan Africa.

We analysed the case-studies against criteria selected to illuminate how research is, or is not, translated into practice in Sub-Saharan Africa. Our analysis centres on the relationships between researchers, policy makers and practitioners (collectively referred to as ‘actors’) in the region.

## Methods

The selected case-studies were presented at a meeting on research engagement with policy and practice in SRH and HIV, which involved researchers, communication specialists and donors working with DFID-funded SRH and HIV Research Programme Consortia. One of the aims of the meeting was to explore and discuss strategies to influence uptake of research into policy and practice in SRH and how context relates to those strategies? Our analysis has been informed by key themes emerging from the meeting, and discussions between all authors. The criteria against which the case-studies were chosen illustrate the role of partnerships, networking and interaction of researchers, with both policy and practice actors in SRH research in Sub Saharan Africa:

Criteria 1: The role of research, advocacy and engagement – exploring policy barriers to change in SRH practice. Two case-studies were chosen against these criteria as summarised in Table 1.

**Table 1:** Criteria 1

Case-study	Facilitator of research uptake	Impediment to practical application
CS1. Maternal syphilis screening. Ghana.	Screening policy introduced as a result of medical evidence, existence of international guidelines and stakeholder partnership.	Limited uptake of syphilis screening due to a) poor dissemination and implementation support, and b) lack of belief in the policy, until local practitioners were consulted.
CS2. Influencing legislative change for sexual violence survivors. Ghana.	Advocacy and networking of a neglected issue was brought to the attention of parliament and resulted in legislative change.	Health care professionals were unaware of new legislation and practical issues prevented implementation.

Criteria 2. The role of developing research to policy networks which act on new research evidence –discussing strategies to build partnerships with policy actors and practitioners. Two case-studies were chosen as summarised in Table 2.

**Table 2:** Criteria 2

Case-study	Role of research evidence	Partnership approach
CS3. Introducing a national adult male circumcision (AMC) policy. South Africa.	Strong local and international evidence coupled with existing cultural practice of AMC used to reconcile traditional and conventional positions.	Researchers used as intermediaries between a national body on SRH & HIV policy and traditional practitioners suspicious of the new AMC policy.
CS4. Scaling up adult male circumcision (AMC) for HIV prevention. Tanzania.	Government convinced of the medical evidence supporting AMC providing that appropriate practice could be ensured.	Stakeholders and practitioners were encouraged to contribute and develop a culturally apt implementation strategy.

These four case-studies were interrogated using Sumner et al’s synthesis approach [1] and Nutley et al’s research use continuum [4] to identify lessons and the dominant types of research use. Sumner’s framework describes the interrelation of three dimensions through which to understand the process of transferring research evidence into policy and/or practice: 1) political context and institutions; 2) policy actors and networks; 3) policy ideas (for example, use and communication of evidence). The details of each case-study are structured according to these three elements. The order in which the elements are presented varies in response to the chronology of events in different contexts.

### Case-study One (CS1). Syphilis Screening and Treatment during Pregnancy in Ghana

Yaw Adu-Sarkodie and Baafuor Kofi Opoku. School of Medical, Sciences, Kwame Nkrumah University of Science and Technology, Kumasi, Ghana

**Background:** Syphilis is easily diagnosed and can be effectively and cheaply treated with antibiotics. Syphilis is known however to be associated with adverse perinatal events, affects between 4% and 15% of pregnant women in Africa and is an important global public health issue. The World Health Organization (WHO) recommends screening during pregnancy and treating infected women with penicillin. Data pertaining to syphilis in pregnancy in Ghana remain either conflicting or unavailable. There is a long-standing syphilis screening policy for pregnant women in Ghana but anecdotal evidence from practitioners suggested that the policy has not been widely practiced.

**Policy context and institutions:** A research agenda, “Maternal Syphilis Screening in Ghana” was developed with the participation of a research team from the Kwame Nkrumah University of Science and Technology and the London School of Hygiene and Tropical Medicine. The research agenda was developed with the DFID-funded RPC for Sexual and Reproductive Health and HIV. Policy makers from the Reproductive Health Unit and the National AIDS Control Programme of the Ghana Health Service, and health staff of user facilities in the country who would implement the findings of the research were also involved. The research assessed the development of the current policy on syphilis in pregnancy and difficulties in its implementation; how much syphilis is seen in pregnant women; the impact on pregnancy outcomes of recent increases in maternal syphilis; existing laboratory diagnostic procedures; and the use of rapid point of care testing in antenatal syphilis screening and potential impact in reducing neonatal deaths.

This piece of operational research was largely carried out in health facilities by existing staff, consequently improved research capacities were built in to those facilities. Research data were gathered from over 200 sites. They found that practitioners had not been implementing the maternal syphilis screening protocol, were unaware of the policy and had not been issued with the equipment required to undertake screening. The institutional context in Ghana has a ‘top down’ approach; in this case the administrative structure resulted in a break-down in communications between the policy level and practitioners at health facility level.

**Policy actors and networks:** Stakeholders expected to have findings reported back to them and remained proactive throughout the process; they were dynamic participants in the dissemination discussions that took place in both formal and informal surroundings. Senior members of the research group took a facilitation role between policy and programme staff who could deliver solutions, and implementation staff who could identify barriers at facility level. A central function of the researchers, who were longstanding colleagues with senior members of the Ghana Health Service, was therefore as communicators between policy and practice levels. Policy makers from the Reproductive Health Programme appeared to be more receptive to accepting the credibility of research findings collected by facility level health staff and trusting evidence presented by known colleagues with whom they had existing relationships than they would have been of external researchers.

**Policy ideas and evidence:** Important reasons given by staff for not following the policy were poor dissemination of the policy to health facilities, insufficient guidance for staff to implement the strategy, logistical problems and lack of belief in the importance of the policy. Policy makers understood the evidence and recognised the importance of following through the evidence-based policy to practice. In this setting the development of a strategy to communicate the research evidence was therefore found to be less relevant than improving existing links within the health sector to translate the existing policy to a recognised and practicable programme.

**Outcomes:** Two strategies were suggested as a result of the research findings in order to strengthen the maternal syphilis screening policy and help ensure its future implementation: incorporation of syphilis screening and treatment into i) the existing prevention of mother-to-child transmission (PMTCT) of HIV programme, and ii) the free maternal health programme. National roll out of maternal syphilis screening at prenatal clinics using rapid point-of-care diagnostic tests has been initiated, and real progress observed with staff training at facility level and the basis to roll out a functioning maternal syphilis screening nationally. The researchers believed that the involvement of all stakeholders throughout research planning and design, conduct of research and result dissemination gave them ownership of the research and thus increased the likelihood of policy adaptation and implementation. Future challenges lie at programme level rather than in syphilis screening practice per se. Accountability for the maternal screening programme is shared between the maternal health programme (with the greater responsibility but proportionally less funds) and the NACP/STI programme (smaller responsibility but with supplementary financing by external donors). The correct allocation of funds and coordination between the two programmes may remain contentious

### Case-study Two (CS2). Influencing Legislative Change for Sexual Violence Survivors In Ghana

Nana Oye Lithur. Human Rights Advocacy, INDEPTH network, Ghana.

**Background:** In 2008 the INDEPTH research network in Ghana commissioned an assessment of the laws and policies governing SRH; the work, undertaken by a high profile human rights lawyer, showed that there were deficiencies in laws and that they were not high priorities for government officials. Legislation and policies regarding sexual violence were of particular cause for concern with the research revealing important gaps in policy and insufficient protocols. Important findings in the assessment included evidence that many victims of sexual abuse and domestic violence survivors could not afford the fee they were required to pay for medical reports and examinations. Non-payment of the fee meant that those cases could not be prosecuted; that some health facilities refused to treat survivors who had not been referred to them by the police; and that survivors were unable to access the few post-traumatic stress services available. The study also revealed that HIV post-exposure prophylaxis was not offered to those survivors who did present themselves at health care facilities, thereby exposing them to the risk of HIV infection. Changes in the legislation on sexual abuse and domestic violence reporting would be required to address the barriers faced by survivors.

**Policy actors and networks:** A strategy was devised to share findings of the assessment with key stakeholders: they were shared widely with parliamentarians, regional directors of health, social services, the Commission on Human Rights and Administrative Justice and traditional, religious and women leaders, in the capital Accra and in two regions. The researchers identified parliamentarians as the key targets of their evidence. Recognising they would be difficult to engage with and would be driven by diverse political interests, dissemination of research findings was adapted accordingly. Direct engagement with parliamentarians was necessary, as was respect for their limited time and the need to present them with clearly articulated approaches with which to address the legislative and policy issues. Parliamentarians, particularly those connected to the health sector, were targeted with tailored communication strategies after which meetings were organised by parliamentary clerks at which solutions were presented. The presentations were made by carefully selected and credible message-bearers who were professional, well-respected legal advocates and researchers. At a public level, engagement took place with the media using compelling human stories supported by statistics to generate public interest and to raise the profile of the issues. The media were identified as important allies in complementing the parliamentary lobbying activities as a result of their coverage.

**Policy context and institutions.** The legislature was important in providing an environment in which a comprehensive set of policies and laws that provide for SRH could be developed, and – theoretically - be implemented. Once new legislation was created, however, there were no immediate provisions by policy makers or programme managers to create and disseminate new guidelines to health providers or ensure law enforcement mechanisms were in place. The research team identified that there were insufficient communication strategies, follow-up and monitoring systems to ensure that SRH laws or policies were actually implemented.

**Policy ideas and evidence:** The targeted approach to disseminating research evidence helped to convince politicians of the breadth and seriousness of the issues and encourage decision-making that could address them. Whilst the dissemination of the research assessment was taking place, a Bill on Domestic Violence was also being considered in Parliament. This coincidence created an appropriate opportunity for parliamentarians to amend the proposed domestic violence bill to include a provision that mandates health care providers to provide free medical treatment to survivors of sexual abuse and domestic violence, pending a complaint to the police and the issuance of a report. The clear recommendations were directly related to parliamentary mandate and were easily incorporated in to policy.

**Outcomes:** Legislative change and then inclusion of a provision in the law were instigated. Sexual violence survivors in Ghana should now receive free medical treatment in advance of reporting a sexual violence case to the police. Subsequent feedback to the researchers in 2009 and 2010 suggested that despite the amended legislation there was little practical change: participants in regional workshops stated that fees were still being demanded by health providers who were not aware of the new law; the police continued to issue forms which did not reflect the new Domestic Violence Act to rape victims; health care providers took advantage of the old forms to charge fees for medical reports. There is a need for engagement between policy makers and the Ghana Health Service to develop strategies to ensure the laws on free medical treatment are followed and to address incidences of corrupt practice. To address this challenge a secretariat has been created at the Ministry of Women and Children tasked with the dissemination of the legal amendment, the coordination of its implementation and educational training. On-going engagement between the researchers, advocates and government officials has been instrumental in addressing the disconnect between policy development and practice.

### Case-study Three (CS3). Introducing a National Male Circumcision Policy into South Africa through the South African National AIDS Council

Eugene Sickle and Sinead Delany-Moretlwe. Reproductive Health & HIV Research Unit, University of the Witwatersrand, Johannesburg, South Africa.

**Background:** The incontrovertible evidence in support of adult male circumcision as a strategy for the reduction of HIV transmission means that South Africa has a human rights and ethical duty to develop a national male circumcision policy. (10-12). For a variety of complex reasons translation of research into policy and then into practice has been slow. The initial “research to policy” drive lost momentum because of poor in-country communication of the research, coupled with weak public understanding of, and engagement with, science. Researchers continued to drive the creation of an adult male circumcision (AMC) policy, working with the World Health Organization (WHO) and UNAIDS to recommend it “as a new, additional prevention strategy for HIV prevention in men” (13).

**Policy context and institutions:** The “research to policy” activity was re-invigorated by the reconstitution in 2008 of The South African National AIDS Council (SANAC) as the pre-eminent national advisory body, providing strategic and political guidance to government on issues of policy on HIV/AIDS. SANAC endorsed the National Strategic Plan for HIV/AIDS [13], lists male circumcision as an “add-on” prevention strategy, thereby giving impetus to the development of a national policy. As the creation of a comprehensive male circumcision policy gathered momentum activity was once again stymied by contestation from within SANAC by traditional leaders. SANAC is a body created by the Department of Health; it is a broad forum which represents 17 sectors. As a broadly representative forum, they were seen as an honest broker in the consultations around this policy because they were an inclusive body which sought to get input from a broad range of stakeholders about issues. Researchers as part of this body were in a position to be able to negotiate for policy change as part of a broader grouping that included the Department of Health.

**Policy actors and networks:** For many South Africans, male circumcision is an integral part of culture and an important step in the initiation of boys into manhood. The practice is the domain of traditional leaders and traditional healers within the South African context. Researchers, through SANAC structures, engaged traditional leaders, recognizing that the development of a policy must be respectful of the important roles played by traditional and religious practices regarding male circumcision. This requires an on-going dialogue with traditional leaders and healers and faith-based sectors about what circumcision may mean for amending and improving the practice of traditional interventions as well as for the evolution of effective AIDS prevention strategies.

The translation of research on male circumcision into policy has required key stakeholders to navigate a complex set of relationships and interactions. Researchers have been required to engage at all stages of this process, often beyond their traditional roles. Through broad-based consultation a deeper understanding of the benefits of male circumcision in the context of HIV prevention has been achieved and ultimately, the development of a comprehensive policy has been realised.

**Policy ideas and evidence:** When SANAC took over the policy consultations there was substantial investment of time in stakeholder meetings to explain the scientific rationale for the policy and to reassure concerned groups that this would not infringe on cultural practices or women’s rights. Thus the final policy was framed as a package of interventions around improved sexual and reproductive health. In this setting, science was used to explain policy and was an important aid to stakeholders’ appreciation of the policy. Evidence was presented in a way that tried to explain the hierarchy and strengths of currently available evidence. Dialogue was valuable as it enabled SANAC members and policy makers to receive and respond to feedback and concerns from those groups that did not support male circumcision. Scientists were able to interpret data for stakeholders and work with other like-minded groupings to address concerns in a way that was acceptable. For example, presentations that addressed key concerns of adult male circumcision safety, women’s risk and rights, were conducted. As more evidence emerged it was incorporated into discussions and used to respond to concerns that were raised. The dialogue between SANAC and stakeholders was framed as a rational discussion – the data served to move the discussion away from extreme views and into the realm of what was known or what still needed to be determined. This was important as part of a broader trend to make research and science more accessible to people and to change perceptions that research is not useful.

**Outcomes.** This positive engagement allowed researchers, SANAC and WHO to set a comprehensive policy agenda with input from key figures in civil society, traditional leadership structures and government. It was a collective effort but the participation of scientists in civil-society government structures has facilitated “change from within”. The result was the development of broad-based advocacy and community-messaging campaigns under the auspices of SANAC and constitution of an expert working group tasked with policy development and the National Department of Health embarking on a nationwide situational analysis as to the feasibility of introducing adult male circumcision into the public healthcare environment. The consultations resulted in broad acceptance of the policy and there are roll-out programmes in provinces that traditionally did not circumcise.

The culmination of these activities saw the creation of a comprehensive policy, the aim of which is to improve male sexual and reproductive health, and reduce new HIV infections through the provision of safe, accessible, sustainable and voluntary clinical male circumcision services in South Africa. There are several demonstration sites including one that has been trying to mobilise the whole community for male circumcision. In KwaZulu-Natal King Goodwill Zwelithini, a traditional leader announced and endorsed the male circumcision programme in selected sites. Despite some controversy in this traditionally non-circumcising province, there has been strong political and traditional endorsement of programmes.

### Case-study Four (CS4). Scaling up adult male circumcision for HIV prevention. The Tanzanian experience

Mwita Wambura, Joseph Mwanga, Jackline Mosha, Gerry Mshana, Frank Mosha, John Changalucha. National Institute for Medical Research, Mwanza, Tanzania.

**Background:** HIV/AIDS remains the most important public health problem in Tanzania) [14]. Promoting effective interventions that prevent new infections and control of the epidemic is a policy priority. Adult male circumcision (AMC) is effective in mitigating the acquisition of HIV infection in men [10-12]. The Tanzanian government was keen to introduce AMC on a large scale from 2007, but it was felt that there was limited information on cultural attitudes and practices towards circumcision, the safety of the procedure, techniques used in both clinical and traditional settings and the capability of the existing health service infrastructure to deliver safe male circumcision services.

**Policy context and institutions:** Two AMC relevant policies have been in place for several years, The National HIV/AIDS Policy of 2002 and the Traditional and Alternative Medicine Act of 2002. Rather than formulating a new policy it was agreed to adapt them to accommodate AMC as an intervention against HIV infection. Strong political will to create a forum to develop AMC policy was in existence. In order to provide national leadership, coordination, resource mobilisation and advocacy, the Tanzanian Ministry of Health and Social Welfare (MoHSW,) following WHO guidance, therefore formed two bodies (a Taskforce Committee and Technical Working Group) to oversee the scaling up of circumcision services in the country. These bodies were formed through a consultative and inclusive process involving all stakeholders. Among the decisions taken by the two oversight bodies were designating a focal person at the MoHSW to coordinate day-to-day work, drafting AMC guidelines for health practitioners and using these to train service providers. The two bodies also approved the implementation of a situation analysis of male circumcision in Tanzania and the on-going male circumcision demonstration projects in four Regions. The National Institute for Medical Research (NIMR) had conducted earlier studies on the topic [15,16] and was tasked to carry out the situation analysis to ensure that decisions are based on sound evidence. Its involvement to provide evidence for the policy development process was a natural progression of the previous research work that NIMR had conducted.

**Policy actors and networks:** Momentum was given to the process from policy actors at all levels. The president of the United Republic of Tanzania was enthusiastic for AMC to be introduced as an additional strategy against HIV infection. Members of the oversight bodies were selected from several sectors, their selection based on, amongst other criteria, experience in policy formulation, implementation and advocacy issues and technical competence in AMC issues. Actors with a range of expertise contributed, including those from the donor community, NGOs, policy makers, researchers, advocacy groups and AMC practitioners in the policy process all of whom were crucial in the scale up efforts The lobby and advocacy group led the advocacy and mobilisation campaigns, the donor community funded the situation analysis study through the government of Tanzania and funded the development of a costed action plan and AMC demonstrations sites. Researchers conducted the situation analysis study, whilst AMC demonstration sites are staffed by AMC practitioners and NGOs. The introduction and scaling-up of AMC services exemplifies the necessity of co-ordinated action from multiple stakeholders. The Tanzanian experience of planning and rolling out male circumcision services illustrates the potential of an inclusive, interconnected and on-going relationship required between policymakers, donors, advocacy groups, researchers and AMC practitioners within a national health programme.

**Policy ideas and evidence:** The policy development process started after evidence that emerged from trials conducted in South Africa, Kenya and Uganda showed that AMC provided partial protection against HIV infection. There was however a lack of locally relevant information required to inform the policy development process. Locally specific research was therefore undertaken as part of the situation analysis study; local research findings informed the development of the Tanzanian strategy and the planning of AMC demonstration sites. Experience gained from demonstration sites further informed the policy features such as ensuring that services are affordable; and defining the minimum quality of care provided to those who undergo AMC as an intervention against HIV infection. Findings from the situation analysis showed a high level of acceptability of male circumcision in both traditionally and non-traditionally circumcising populations. Traditional leaders play a key decision making role in circumcision and should have a role to play in the national strategy for the national programme to be successful in traditionally circumcising areas. Health systems need strengthening for effective delivery of circumcision services. NIMR presented these findings to the two oversight bodies and an implementation strategy was developed.

**Outcomes:** AMC scale-up is under-way and there is enabling environment regulated by policy. New policy was not developed but existing policies were adapted to include provision of safe male circumcision procedures for the prevention of HIV infection. Several challenges remain such as the integration of traditional and clinical based circumcisions and how limitations within the public health system will affect the national AMC programme.

## Results and discussion

Sumner et al’s synthesis approach [1] suggests that the three meta-domains of policy change (policy actors or networks; policy ideas and evidence; and policy context or institutions), used here to frame the case studies, can be divided into two categories determining policy outcomes. These are 1) the ‘pre-conditions’ for research use (meaning the existing policy context), and 2) actions and interventions (or those things which increase the probability of research being used by policy actors). We present the salient issues arising from these case-studies in terms of what have been useful lessons in shaping policy uptake and/or practice. Within this approach we identified the importance of how research, policy and practice actors communicated and interacted with each other. One of the most palpable lessons is the diversity of actors involved in policy and practice and both the importance and complexity of on-going and continuous communication with all actors. This requires investing in knowledge exchange: money and time, people, and capacity to communicate research sensitively and appropriately within the context.

**Policy actors and networks.** All four case-studies presented here demonstrate conceptual research use with – sometimes subtle – communications strategies supporting the creation of policy and practice networking and links. In these different settings the approaches encompassed multi-stakeholder partnerships including policy and practitioner communities, intermediaries, advocacy communities and traditional leaders in order to influence attitudes and re-enforce the power of the research evidence and knowledge that was to be applied to a given policy or practice.

CS1, CS3 and CS4 incorporated practitioners into their programmes, and recognised their policy-influencing potential from the outset; in CS3 and CS4 the on-going dialogue, including representatives of traditional practices, resulted in the design of adult male circumcision policy with some understanding of the subtle cultural and technical needs of different actors. In the Ghana case-studies (CS1 and CS2) a distinction in the role of policy actors is apparent that illustrates the potential efficacy of engagement. In both settings researchers identified existing SRH policy which was not followed and were able to mobilise change at policy level; however benefits in practice have been less clear. CS1 (maternal syphilis screening in Ghana) was able to redress a failing policy by using an individual – the researcher from a respected academic institution who had close ties with the Ghana Health Service – to act as an intermediary between the policy makers and health practitioners which appear to have had positive practice outcomes. A comparable situation occurred in CS2 where government action was not followed through to implementation; the research communications strategy was effective and resulted in the desired legislative change, yet this was not sufficient to ensure change in health service practices. Renewed engagement was later seen to be necessary to address the institutional bottleneck.

**Policy ideas and evidence.** The relevance and credibility of research evidence is important, but it also needs to be operationally appropriate and practicable. Researchers were aware of the fallibility of the policy processes, they were sensitive and reacted innovatively; policy makers in all the case studies therefore responded well to research evidence pitched to them. Evidence was designed to address political concerns and local situations, and was done in appropriate ways in all four contexts. The male circumcision case-studies (CS3 and CS4) illustrate that while the evidence suggested solutions to a recognised problem (HIV transmission) policy makers were not convinced of the political opportunity to introduce policy just on the back of strong research evidence on an intervention so shrouded in controversy (particularly in South Africa) as circumcision. In the case of maternal syphilis screening (CS1), evidence-based international guidelines which had guided the existing policy were not followed. In these three cases conceptual issues needed to be addressed such as knowledge gaps (CS1, CS3), communicating science in an accessible way (CS1, CS3, CS4), creating a rights based approach (CS3) and local suitability (CS3, CS4) in addition to the technical implementation challenges (CS1,CS3,CS4). In these circumstances evidence alone may not have been strong enough to have an impact on policy and investing in multi-stakeholder dialogue was effective in discussing how research evidence could be applied to a culturally appropriate and safe practice in three cases. CS2 (Sexual and Domestic Violence in Ghana) illustrated that while evidence was persuasive to parliamentarians, it was only when researchers later re-assessed the impact of the new laws by contacting practitioners that it became clear that health practices had not changed.

**Policy context and institutions.** Policy context refers to the underlying institutional and political situation and to some extent external institutional influence on policy and practice from, for example, donor organizations or the WHO. The intrinsic sensitivity of issues on SRH and HIV which reflect the moral, religious and cultural make-up of a context can result in conflict between institutions or reluctance to deal with controversial topics. Policy context is arguably the most important pre-condition for the degree to which research evidence is initially taken up; it is also sensitive to conceptual research use. These case studies demonstrate how local researchers and policy actors applied their knowledge to try to ensure political institutions and policy actors were responsive to research evidence. Creating new partnerships between existing institutions occurred in all cases, including between the health sector, the media, the legislature, community groups, traditional leaders, advocates and policy makers. Involving traditional practitioners and community groups in partnerships was vital in developing AMC strategies (CS3 CS4); linking policy makers with practitioners was central to success in developing maternal syphilis screening rollout; in contrast CS2 (Ghana), researchers first targeted individuals in order to address legal limitations and there were no existing mechanisms for dialogue between high level officials and practitioners. These case-studies have illustrated the importance of the institutional and political structures in influencing the willingness of policy actors to act on new research and particularly the importance of forging collaborative relationships between relevant institutions. Establishment of new institutional structures in the male circumcision case-studies (CS3, CS4) facilitated uptake of research evidence into policy.

The case-studies in Ghana (CS1, CS2) illustrated institutional willingness to incorporate new evidence into policy amendments, but both cases show limitations in the policy dissemination process. In CS2 despite success in health legislation, there has been subsequent recognition that practitioners were unaware of this. A similar situation had occurred in Ghana (CS1) prior to the maternal syphilis research study described here, the research team’s response was to make an assessment of policy compliance as part of their research design and the multi-stakeholder approach created new institutional links to overcome barriers to maternal syphilis screening. Three of the case studies highlighted that policies were not being implemented on the ground, highlighting the potential for undertaking a process of engagement with practitioners and creating mechanisms for follow-up as part of the research use continuum.

Many of the existing frameworks for analysing how research evidence may be translated in to policy and practice acknowledge that it involves a combination of overlapping processes relating to the context, research evidence and networks outlined here. The four case-studies demonstrate a distinction between research that is used in policy, and research which is then also translated into practice.

Our case-studies illustrate that working simultaneously at both instrumental and conceptual levels, whereby stakeholders are involved throughout the process, is important in ensuring that practice can receive comparable priority to policy. Conceptual uses of research evidence are vital contributions to the ultimate policy goal of practical application because they can work on many layers, engagement is capable of altering the attitudes and knowledge of those who are instrumental in both policy and practice. The source of research evidence, its expected audience in the policy arena and the optimal policy uptake of evidence, are all important considerations when initiating action with stakeholders and developing strategies for communicating evidence. Conceptual research use is difficult to measure. However we can demonstrate that programmes and policy agendas that fail to consider *conceptual* use of research can have difficulties in achieving successful outcomes of *instrumental* research use in health practices. The contrast in our two types of case-studies is a pertinent illustration of this. We now go on to show the importance of this distinction in relation to the case-studies on adult male circumcision.

There has been a rapid accumulation of academic literature and print media as a result of the mixed and deep-rooted feelings that evidence regarding male circumcision trials has produced (see Table 3). There is strong evidence that male circumcision can reduce heterosexual acquisition of HIV by between 51 and 60% [10-12] and studies have suggested there is a high level of acceptability (67%) of AMC by many men from those parts of sub-Saharan Africa where circumcision is not the norm [17]. The concept of male circumcision is embedded in culture and image of ‘the self’; it is an important part of individuals’ lives and identities, yet it is also associated with misconception, immense controversy and varied attitudes between communities, ethnicities, religions, health practitioners and policy-makers globally. Debate on male circumcision has shown the potential for misunderstanding and conflation with female genital mutilation (FGM), for example a Ugandan study identified that some people consider male circumcision a justification of FGM [18], these findings confirm that circumcision messaging and social context and gender issues need particularly sensitive handling. Table 3 summarises the key debates on male circumcision in sub-Saharan Africa, debates that suggest controversial and highly emotive subjects might have a significant impact on whether a policy is enacted, how it is implemented and how its implementation affects service users and service providers. The debates demonstrate the considerable array of issues which would we saw benefitted from a conceptual use of research evidence throughout the continuum. Conceptual research use to create accurate messaging that provides knowledge and understanding on a controversial topic is vital on many levels: 1) persuading political actors of the relative costs, risks and benefits of providing male circumcision in their context; 2) advocating to traditional and clinical practitioners about the validity of male circumcision as a preventative measure and how to apply this prevention intervention to their populations in a safe and ethical manner; 3) providing relevant and consistent information to the target population. The instrumental direct use of the research evidence, that is, evidence that proves the protective effect of male circumcision, can be applied appropriately only when the conceptual issues around this controversial intervention on different stakeholders’ diverse and varied views and beliefs have been addressed. (Table 3)

**Table 3:** Key debates in the literature on adult male circumcision (AMC) evidence, policy and practice in Sub Saharan Africa

Type of debate	Challenge
Social, cultural and religious factors	Is AMC policy culturally acceptable in this context? What are the current traditional or ritualistic practices surrounding AMC? Are there gender specific risks (i.e. FGM conflation)?
	
	Have risks, benefits and harm reduction been taken in to account for the social and cultural and geographic specific factors of this setting?
	
	Is there willingness for adult males to be circumcised and is it acceptable to circumcise male children?
	
	Can it be made clear to the public that AMC policy is being introduced as one part of a combination approach to prevention?
	
	How can voices reflecting the socio-cultural context be heard and inform AMC policy and practice?

Messaging	Can clear and consistent communications strategies be devised that clearly demonstrate that protection from HIV is relative and not absolute?
	
	How can misunderstanding be minimised, so, for example male circumcision and female genital mutilation will not be conflated
	
	Is it possible to create clear understanding on the importance of abstaining from sex until the wound is healed.
	
	How can men and women be educated to avoid ‘risk compensation’ whereby women are at risk by circumcised men not agreeing to safe sex, or men attracting (through their new status) a large number of female partners?
	
	Will AMC also protect women (through a reduction in HIV and STI incidence among men) or increase their risk of HIV infection due to disinhibition of their male partners?
	
	Will provision be required to prevent conflation with FMG in this context?

Provider issues	Are there sufficient trained and knowledgeable medical personnel and sterile instruments in this setting?
	
	Will this policy create a strain on the health systems, potentially at the expense of other important interventions?
	
	How will traditional male circumcision techniques be regulated to encourage safe, correct practice and prevent their higher reporting of adverse events?
	
	Will human rights and ethical principles of consent be adhered to?

There are commonalities with these debates and other SRH and HIV policy and interventions due to the inherently sensitive nature of creating policy and changing practice which affects the most private spheres of people’s lives.

In relation to policy change in Ghana, research use that emphasised a two-way flow of information benefited policy change whereby researchers could orientate findings to targeted policy actors and research users could adapt findings to their specific contexts. Conceptual research use that focuses on forging partnerships was seen to facilitate understanding and knowledge acquisition between research, policy and practice actors. The parliamentary reform case-study from Ghana (CS2) suggested that the positive outcomes were possible despite the controversial nature of the subject and the low priority this area has traditionally had; change occurred as a result of targeted advocacy, the use of a clear policy window and instrumental use of research evidence. Researchers have undertaken long-term follow-up and dialogue with officials to address the institutional challenges preventing changes in police and health practices. The maternal syphilis screening case-study demonstrated that in the past a similar problem of low practical uptake of policy had prevailed. An implementation strategy was considered only retrospectively, at which point a more conceptual research use was adopted and consultation with practitioners themselves allowed solutions to be proposed and introduced.

## Conclusion

The case study analysis suggests that there is a real need to think about the practical implementation of a policy from the inception of a programme; we need to move beyond conceptualising a linear process that begins with conceptual use of research evidence (tackling attitudes and knowledge) and finishes with instrumental and attributable use.

Creating strategies for communicating research evidence with SRH practices in mind has been core to the analysis of what approaches have been successful. The role of leadership and support in research communications is highlighted by Nutley et al [19] in a cross-sector review on promoting evidence based practice. Co-ordination and partnerships can encourage greater understanding, change attitudes and have an impact on practice and policy implementation, while dedicated financial resources for communications are vital in ensuring these subtle influences occur (see South this supplement, [20]). Nutley et al suggest [4] that evidence-based practices are at risk of over emphasising the instrumental use of research evidence at the expense of conceptual use, without which we cannot expect to grasp how research can influence understanding and receptivity to evidence. Politicians are influenced by a great many frequently competing factors, of which research evidence may not be the most significant. Health initiatives that become prioritised are not necessarily those associated with the greatest levels of morbidity or mortality, rather, those which have been effectively positioned by policy communities [21].

The linkages and networks between stakeholders and particularly the central role of practitioners that occurred in the case-studies suggest the significance of designing networks and partnerships carefully from a project’s inception. Participatory approaches and engagement strategies directed by well-connected local individuals were seen to have strong impacts in the contexts scrutinized here. Researchers and communication specialists need to work at developing networks of actors across the policy and practice continuum, while designing long-term communications strategies appropriate to a range of specific technical, political and cultural contexts. This may involve working simultaneously at conceptual and instrumental levels to connect researchers with both policy makers and practitioners.

## Competing interests

This article critically reflects on research projects that the following authors have been involved in: YAS, NOL, ES, WM, SDM, PM, JC, BKO.

## Authors’ contributions

The writing of each case-study included in this paper was led by an individual from each research programme represented: Yaw Adu Sarkodie, Nana Oye Lithur, Eugene Sickle, Mwita Wambura and included contributions from other key members of their research teams who are acknowledged in each case-study. The main manuscript was drafted by Olivia Tulloch and Sally Theobald, revised by Philippe Mayaud and reviewed by all case-study authors.

## Additional information

Ethical clearance:

The paper is not a specific research study and separate ethical clearance was obtained by the various groups mentioned in the case-studies as follows.

**CS1 Maternal syphilis screening in Ghana:** Ethical clearance was obtained by the Committee on Human Research, Publications and Ethics, Kwame Nkrumah University of Science and Technology, School of Medical Sciences and Komfo Anokye Teaching Hospital, Ghana.

**CS2 Legislative change in Ghana:** No ethical clearance required.

**CS3 Male circumcision South Africa:** Research findings mentioned come from studies conducted by other groups and which have been funded and have obtained ethical clearance separately. Readers are referred to the original papers (mentioned in references) to obtain details of funding and ethical clearance.

**CS4 Male circumcision Tanzania:** The ethical clearance for the study was obtained from the National Institute for Medical Research (NIMR), Mwanza Centre Scientific Committee and the Medical Research Coordination Committee (MRCC) of the National Institute for Medical Research, Tanzani.

## References

[B1] Sumner A Crichton J Theobald S What shapes research impact on policy: Understanding research uptake in SRH policy processes in resource poor contexts Health Research Policy and Systems This supplement 10.1186/1478-4505-9-S1-S3 PMC3121134 21679384

[B2] Court J Young J Bridging Research and Policy: Insights From 50 Case Studies ODI Working Paper 2003 213 London: Overseas Development Institute

[B3] Walt G Gilson L Reforming the health sector in developing countries: the central role of policy analysis Health Policy and Planning 1994 9 4 353 370 10.1093/heapol/9.4.353 10139469

[B4] Nutley S Walter I Davies H Using evidence: How Research can inform public services 2007 Bristol, UK: The Policy Press

[B5] Crewe E Young J Bridging research and policy: context, evidence and links 2002 London: Overseas Development Institute

[B6] Court J Bridging Research and Policy on HIV/AIDS in Developing Countries 2004 London: Overseas Development Institute

[B7] Dickenson C Buse K Understanding the politics of national HIV policies: the roles of institutions, interests and ideas Technical Approach Paper 2008 HSLP. London

[B8] Gilson L Raphaely N The Terrain of Health Policy Analysis in low and middle income countries: a review of published literature 1994-2007 Health Policy and Planning 2008 23 5 294 30 10.1093/heapol/czn019 18650209 PMC2515407

[B9] Greene D David JL A research design for generalizing from multiple case studies Evaluation and Program Planning 1984 7 1 73 85 10.1016/0149-7189(84)90027-2

[B10] Auvert B Taljaard D Lagarde E Randomized controlled intervention trial of male circumcision for reduction of HIV infection risk: the ANRS 1265 Trial PLoS Medicine 2005 2 11 e298 10.1371/journal.pmed.0020298 16231970 PMC1262556

[B11] Gray H Kigozi G Serwadda D Male circumcision for HIV prevention in young men in Rakai, Uganda: a randomized trial The Lancet 2007 369 657 66 10.1016/S0140-6736(07)60313-4 17321311

[B12] Bailey RC Moses S Parker CB Male circumcision for HIV prevention in young men in Kisumu, Kenya: a randomized controlled trial The Lancet 2007 369 643 56 10.1016/S0140-6736(07)60312-2 17321310

[B13] Ministry of Health South Africa National Strategic Plan for HIV/AIDS 2007 – 2011 2007 Pretoria

[B14] UNAIDS/ WHO AIDS epidemic update 2007 Geneva: WHO Accessed on 10.09.2009 from : http://data.unaids.org/pub/EPISlides/2007/2007_epiupdate_en.pdf

[B15] Urassa M Todd J Boerma JT Hayes R Isingo R Male circumcision and susceptibility to HIV infection among men in Tanzania Aids 1997 11 73 9 9110078 10.1097/00002030-199701000-00011

[B16] Nnko S Washija R Urassa M Boerma JT Dynamics of male circumcision practices in northwest Tanzania Sexually Transmitted Diseases 2001 28 214 8 11318252 10.1097/00007435-200104000-00005

[B17] Westercamp N Bailey RC Acceptability of male circumcision for prevention of HIV/AIDS in sub-Saharan Africa: a review AIDS Behaviour 2007 11 3 341 55 10.1007/s10461-006-9169-4 PMC1847541 17053855

[B18] Women’s HIV Prevention Tracking Project (WHiPT) Making Medical Male Circumcision Work for Women 2010 http://www.avac.org/ht/d/sp/d/sp/i/306/pid/306

[B19] Nutley S Walter I Davies H Promoting Evidence-based Practice: Models and Mechanisms From Cross-Sector Review Research on Social Work Practice 2009 19 5 552 559 10.1177/1049731509335496

[B20] South A Designing and implementing a communications strategy: lessons learnt from HIV and Sexual and Reproductive Health Research Programme Consortia Health Research Policy and Systems This supplement 10.1186/1478-4505-9-S1-S15 PMC3121132 21679382

[B21] Shiffman J A social explanation of the rise and fall of global health issues Bulletin of the World Health Organization 2009 87 608 613 10.2471/BLT.08.060749 19705011 PMC2733265

